# Characteristics and outcome of influenza-associated encephalopathy/encephalitis among children in China

**DOI:** 10.1016/j.clinsp.2024.100475

**Published:** 2024-08-02

**Authors:** Min Yang, Ling Yi, Fenglin Jia, Xiaobin Zeng, Zhongqiang Liu

**Affiliations:** a Department of Pediatric Intensive Care Unit, West China Second University Hospital, Sichuan University, Chengdu, Sichuan, China; b Key Laboratory of Birth Defects and Related Diseases of Women and Children, Sichuan University, Ministry of Education, Chengdu, Sichuan, China; c Department of Medical Record Management, West China Second University Hospital, Sichuan University, Chengdu, Sichuan, China; d Department of Radiology, West China Second University Hospital, Sichuan University, Chengdu, Sichuan, China; e Medical Equipment Department, West China Second University Hospital, Sichuan University, Chengdu, Sichuan, China

**Keywords:** Children, Encephalitis, Encephalopathy, Influenza, Characteristics

## Abstract

**Background:**

Influenza-Associated Encephalopathy/Encephalitis (IAE) is characterized by high incidence and poor prognosis. The aim of this study is to describe the clinical features and outcomes of IAE in pediatric patients.

**Methods:**

We performed a retrospective review of hospitalized cases of laboratory-confirmed influenza infection between January 2018 and December 2021. Demographic, clinical, imaging, treatment and outcome data were collected. Statistical analysis was performed using SPSS software.

**Results:**

Of 446 children hospitalized with influenza, 71 cases were identified with a diagnosis of IAE. The median age was 3 years and 46 (64.8 %) were younger than 5 years. Only one patient was vaccinated for seasonal influenza. 46 (64.8 %) patients had abnormal electroencephalogram examination and 47 (66.2 %) had abnormal brain MRI or CT findings. 68 (95.8 %) patients were treated with oseltamivir/peramivir. 12 (16.9 %) patients suffered mortality. Non-survivors were more likely to have lower Glasgow coma score (median 7), longer duration of fever (median 3 days), with underlying medical conditions (*P* = 0.006), and complications including sepsis (*P* = 0.003), shock (*P* < 0.001), respiratory failure (*P* = 0.006), acute renal failure (*P* = 0.001), myocardial damage (*P* < 0.001), coagulation disorders (*P* = 0.03), electrolyte disturbance (*P* = 0.001) and hyperlactacidemia (*P* = 0.003). Non-survivors had higher percentages of corticosteroids (*P* = 0.003) and immunoglobulin (*P* = 0.003) treatments compared to survivors.

**Conclusions:**

Children with IAE have a high mortality rate. Lower Glasgow coma score, longer duration of fever, with underlying medical conditions and complications pose a great risk to poor prognosis. Influenza vaccination is recommended to all eligible children.

## Highlights

• Retrospective review of hospitalized IAE cases in a large tertiary pediatric hospital (National Children's Regional Medical Center in China) which admits over 15000 pediatric patients every year.
• Lower Glasgow coma score, longer duration of fever, with underlying medical conditions and complications pose a great risk to poor prognosis.
• Children with IAE have high mortality rate. Influenza vaccination is Recommended to all eligible children.

## Introduction

Influenza infection primarily causes respiratory illness and has a substantial burden of hospitalizations in children around the world.^1^, ^2^, ^3^, ^4^, ^5^ The estimated incidence of influenza in the United States was 8.7 % for children during 2010–2016.^2^ Japanese administrative data revealed children aged 0‒5 years were at high risk of hospitalization.^3^ According to statistics from the Chinese Center for Disease Control and Prevention,^6^ the average influenza-like-illness burden was 4.5 consultations per 1000 person-years among children aged below 15 years old from 2006 to 2015, which was almost twice the rate in elderly patients. In addition to respiratory symptoms, neurologic complications including seizures, encephalitis, encephalopathy, focal neurologic deficits, Guillain-Barre syndrome, acute disseminated encephalomyelitis, transverse myelitis, Reye syndrome may also occur in influenza virus infection.^7^^,^^8^ Influenza-Associated Encephalopathy/Encephalitis (IAE) is a serious neurological complication of influenza infection. It was reported in the late 1990s in Japan ^9^ and usually manifested as Acute Necrotizing Encephalopathy (ANE), which has high mortality.^10^

The majority of reports on IAE have been from Japan.^11^^,^^12^ Available literature data about characteristics and outcomes of IAE in children remains limited in other countries. Therefore, we performed a retrospective study on children < 18 years old with influenza infection and diagnosed with IAE. This study aimed to evaluate the clinical characteristics of IAE in hospitalized pediatric patients in China.

## Methods

### Study subjects

We performed a retrospective review of hospitalized patients with laboratory-confirmed influenza infection from January 2018 to December 2021 at West China Second University Hospital (National Children's Regional Medical Center in China), a tertiary pediatric hospital in Chengdu, China, which admits over 15000 pediatric patients every year.

Influenza infection was diagnosed by influenza rapid antigen test or Reverse Transcription-Polymerase Chain Reaction (RT-PCR). All the medical records of laboratory-confirmed influenza cases were reviewed to identify the occurrence of IAE. The diagnosis of IAE relies on criteria recommended by ‘Guidelines for the Diagnosis and Treatment of Acute Encephalopathy in Childhood’.^13^ All IAE cases were confirmed by 2 experienced pediatricians. Demographic, clinical, laboratory, radiological, treatment and outcome data were extracted from patients’ medical records. We used the STROBE Statement checklist when writing this report.

### Statistical analysis

Continuous variables were expressed as median and Interquartile Range (IQR). Categorical variables were expressed as number and percentages. Comparison of continuous variables was carried out by using the Mann-Whitney *U* test. Comparison of categorical variables was analyzed by using the χ^2^ test or Fisher exact test; *P* < 0.05 was defined as statistically significant. Statistical analysis was performed using SPSS version 21.0.

### Ethics statement

This study was approved by the Ethics Committee of West China Second University Hospital (Approval Number: 2020-111). The informed consent of patients was waived since only retrospective aggregated data analysis of medical records was involved. The present data were fully de-identified and anonymous to protect privacy.

## Results

Of 446 children hospitalized with laboratory-confirmed influenza infection from January 2018 to December 2021, 71 (15.9 %) cases were detected with a diagnosis of IAE. IAE in children mostly occurred from October to March. There was a corresponding increase with the outbreak of the influenza epidemic year.

General characteristics are summarized in Table 1. Of all IAE patients, the median age was 3-years (IQR 1–5). 46 (64.8 %) patients were less than 5-years old and 48 (67.6 %) patients were male. 45 (63.4 %) patients were positive for influenza A and 23 (32.4 %) patients for influenza B, with 3 (4.2 %) patients showing positive results for both strains. Only one patient received seasonal influenza vaccination. 46 (64.8 %) patients had underlying medical conditions, including cerebral palsy or delayed development, seizure history, malnutrition, respiratory diseases and cardiovascular diseases. Of these patients, 6 patients had two underlying medical conditions (1 patient had both cerebral palsy or delayed development and malnutrition, 2 patients had both cerebral palsy or delayed development and seizures, 3 patients had both cerebral palsy or delayed development and malnutrition), and 1 patient had three underlying medical conditions (cerebral palsy or delayed development, malnutrition, and respiratory disease.

**Table 1:** General characteristics of the patients with IAE.

	Number of Cases (%)
**Age**	
< 1 year	9 (12.7 %)
1‒4 years	37 (52.1 %)
5‒10 years	22 (31.0 %)
> 11 years	3 (4.2 %)
**Sex**	
Male	48 (67.6 %)
Female	23 (32.4 %)
**Type of influenza**	
A	45 (63.4 %)
B	23 (32.4 %)
A and B	3 (4.2 %)
**Influenza vaccine**	1 (1.4 %)
**Underlying medical conditions**	46 (64.8 %)
Cerebral palsy or delayed development	20 (28.2 %)
Seizure history	15 (21.1 %)
Malnutrition	12 (16.9 %)
Respiratory diseases	4 (5.6 %)
Cardiovascular disease	2 (2.8 %)

Clinical features are presented in Table 2. Most patients had a fever (88.6 %) and a seizure (80.3 %). 41 (57.7 %) patients had a Glasgow coma score of no more than 13 scores. 40 (56.3 %) patients had abnormal physical signs of nervous system, of which 10 patients had underlying medical condition of cerebral palsy. However, these abnormal signs were not specific to IAE patients. 64.8 % of the cases had abnormal findings of Electroencephalogram (EEG) examination, mostly showing slow-wave activity. Brain Magnetic Resonance Imaging (MRI) or Computed Tomography (CT) was performed in 65 cases and revealed abnormalities in 72.3 %. Typical characteristics of ANE were found in 8 cases, with manifestations of symmetrical and multiple brain lesions. Bilateral thalamic lesions were always observed. Lesions were also found in periventricular white matter, internal capsule, putamen, upper brainstem tegmentum, and cerebellum. Other cranial imaging findings of children with IAE showed a variety of manifestations, including scattered abnormal signal shadows and edema in the cerebral cortex, white matter lesions in different parts of the brain. Cerebrospinal Fluid (CSF) specimens were obtained from 52 (73.2 %) patients and the findings were normal in most of them.

**Table 2:** Clinical features of the patients with IAE.

	Number of Cases (%)
**Fever**	62 (88.6 %)
**Vomit**	29 (40.8 %)
**Seizure**	57 (80.3 %)
**Glasgow coma score (≤13)**	41 (57.7 %)
**Abnormal physical sign of nervous system**	40 (56.3 %)
**Abnormal pupil size**	7 (9.9 %)
**Abnormal pupil reflexes**	13 (18.3 %)
**Meningeal irritation sign (+)**	15 (21.1 %)
**Pathologic sign (+)**	26 (36.6 %)
**Tendon reflex drops or disappear**	11 (15.5 %)
**Paraesthesia**	5 (7.0 %)
**Abnormal muscular tension**	19 (26.8 %)
**Abnormal of EEG**	46 (64.8 %)
Slow-wave activity	23 (32.4 %)
Abnormal of MRI or CT	47 (66.2 %)
Acute Necrotizing Encephalopathy (ANE)	8 (11.3 %)

As shown in Table 3, 12 (16.9 %) children suffered mortality. All non-survivors had underlying medical conditions. Non-survivors were more likely to have underlying medical conditions than survivors (*P* = 0.006). Non-survivors had long duration of fever (*P* = 0.03) and lower Glasgow coma scores than survival children (*P* < 0.001). The most common complications were pneumonia (60.6 %), respiratory failure (36.6 %) and electrolyte disturbance (32.4 %). Non-survivors were more likely to have complications including sepsis (*P* = 0.003), shock (*P* < 0.001), respiratory failure (*P* = 0.006), acute renal failure (*P* = 0.001), myocardial damage (*P* < 0.001), coagulation disorders (*P* = 0.03), hyperlactacidemia (*P* = 0.003), and electrolyte disturbance (*P* = 0.001). Administration of antiviral drugs of oseltamivir/peramivir showed no significant difference between the two outcome groups. Non-survivors had higher percentages of supporting therapeutics of high-dose corticosteroids (*P* = 0.003) and high-dose Intravenous Immunoglobulin (IVIG) (*P* = 0.003) compared to survivors. There were 4 patients treated with plasma exchange of which only 2 children survived.

**Table 3:** Comparison between survivor and non-survivor patients with IAE.

	Survivor (n = 59)	Non-survivor (n = 12)	p
Underlying medical conditions	34 (57.6 %)	12 (100.0 %)	0.006
**Clinical Features**			
Fever	52 (88.1 %)	11 (91.7 %)	1.00
**Fever (days) (median, IQR)**			
Seizure	1 (0‒2)	3 (1.25‒4)	0.03
Vomit	48 (81.4 %)	9 (75.0 %)	0.69
**Glasgow coma score (median, IQR)**	22 (37.3 %)	7 (58.3 %)	0.30
**Complications**	13 (9‒15)	7 (2.25‒10.75)	<0.001
Pneumonia	34 (57.6 %)	9 (75.0 %)	0.34
Sepsis	9 (15.3 %)	7 (58.3 %)	0.003
Shock	4 (6.8 %)	7 (58.3 %)	<0.001
Respiratory failure	17 (28.8 %)	9 (75.0 %)	0.006
Acute renal failure	2 (3.4 %)	5 (41.7 %)	0.001
**Myocardial damage**	5 (8.5 %)	7 (58.3 %)	<0.001
Coagulation disorders	13 (22.0 %)	7 (58.3 %)	0.03
Hypoproteinemia	4 (6.8 %)	0 (0.0 %)	1.00
Hyperlactacidemia	9 (15.3 %)	7 (58.3 %)	0.003
Electrolyte disturbance	14 (23.7 %)	9 (75.0 %)	0.001
**Treatments**			
Oseltamivir/peramivir	58 (98.3 %)	10 (83.3 %)	0.07
High-dose corticosteroids	16 (27.1 %)	9 (75.0 %)	0.003
High-dose IVIG	16 (27.1 %)	9 (75.0 %)	0.003

## Discussion

IAE is one of the serious complications of influenza which leads to poor prognosis in children, and it has attracted more and more attention from pediatric neurologists. The incidence of IAE varies in reports from different countries. Frankl et al. reported that 4.3 % of children hospitalized with laboratory-confirmed influenza were found complicated with IAE in the hospital of the United States.^14^ Solís-García et al. found that the incidence of IAE was 0.8 % in a tertiary-care hospital in Spain.^15^ A report from three hospitals in Korea detected that 0.6 % of pediatric patients with influenza infection were diagnosed as IAE.^16^ In the present study, the incidence of IAE among 446 hospitalized children with laboratory-confirmed influenza infection was 15.9 %, which was consistent with the study from Italy (13.1 %).^17^ The discrepancy of incidence in different countries may be due to distinct thresholds of patients’ admission.

In the present study, the high-incidence season of IAE was winter and spring which was consistent with the high-incidence season of influenza, especially in the outbreak of influenza epidemic years. Children of all ages were generally susceptible to IAE, especially children under 5 years old, which accounted for 64.8 %. We found that children of lower ages were more likely to have IAE when exposed to influenza virus, which was in accord with previous literature.^3^^,^^18^

Mortality rates of IAE in pediatric patients also vary in different studies, ranging from 8 % to 15 %.^12^^,^^16^^,^^19^, ^20^ In this study, the mortality rate of IAE was 16.9 %, which was comparable with reports from Korea and Australia (15.4 % and 14 %, respectively).^16^^,^^19^ However, the rate was higher than reports from Japan and Hong Kong (8 % and 10.9 %, respectively).^12^^,^^20^ The difference might be explained by the diversity of case definitions and severity of illness at admission. In addition, the mortality rates in Japan and Hong Kong were from population-based studies.

In this study, a high proportion of patients with IAE had a fever, seizure and consciousness changes. There was no significant difference between survivor and non-survivor children in the proportion of fever and seizure. However, non-survivor children had a longer duration of fever. Meanwhile, this study also showed that non-survivor children had lower Glasgow coma scores. It is suggested that a longer duration of fever and lower Glasgow coma score indicate a worse prognosis of IAE. Various neurological physical abnormalities occurred in patients of the study, including paraesthesia, abnormal pupil size and pupil reflexes, positive meningeal irritation sign and pathologic signs, tendon reflex drops or disappearing and abnormal muscular tension. CSF, EEG and cranial imaging are important means for diagnosis of nervous system diseases. In this study, no specific abnormality was found in the CSF. 63.5 % of children's EEG was abnormal, with the main manifestation of diffuse slow-wave activity, which was not different from the EEG findings of patients with other virus infections. Similar to other research reports,^7^^,^^13^ the imaging features of IAE were diverse, including diffuse edema in the cerebral cortex, white matter lesions in different parts of the brain, thalamus, brainstem, basal ganglia lesions, and cerebellar white matter lesions with or without brain edema.^8^ children were diagnosed with ANE, which was characterized by symmetrical multiple lesions.

In the present study, 64.8 % of patients had underlying medical conditions, including cerebral palsy or delayed development, seizure history, malnutrition, respiratory diseases and cardiovascular disease. Similar to previous published studies, children with underlying medical conditions were particularly vulnerable to IAE and resulted in poor outcome.^18^^,^^21^ In this study, all 12 children who died of IAE had underlying medical conditions, while only 57.6 % of survivor patients had underlying medical conditions. It seems that underlying medical conditions may be related to a worse prognosis. It's worth noting that a significant proportion of IAE patients were found accompanied with malnutrition, which was rarely reported in the literatures. As we all know, the present study also found that children who suffered mortality had a higher proportion of sepsis, shock, respiratory failure, acute renal failure, myocardial damage, coagulation disorders, hyperlactacidemia, and electrolyte disturbance. In all, a lower Glasgow coma score, longer duration of fever, with underlying medical conditions and complications pose a great risk to poor prognosis.

At present, there is no specific treatment for IAE. The routine treatment schemes reported in the literature include controlling seizures, reducing encephalic pressure and hydrocephalus, and antivirals. In the present study, most patients were treated with oseltamivir/peramivir. However, there was still a high proportion of deaths. Thus, the positive effect of oseltamivir/peramivir in reducing the neurological complications of influenza and improving prognosis is unclear. Recently, corticosteroid, IVIG, and plasma exchange were reported to treat patients with IAE.^22^, ^23^, ^24^, ^25^, ^26^, ^27^, ^28^ Several researches detected that early immunomodulatory treatment including high-dose corticosteroids ^22^^,^^23^ and IVIG 23, 24 can improve the neurological results of influenza-associated ANE in children. But Zhu HM et al.^25^ found that steroid therapy and IVIG had no correlation with better outcomes. These findings showed that non-survivors had higher percentages of supporting therapeutics of methylprednisolone and IVIG, which indicates that corticosteroids and immunoglobulins may not be directly related to good prognosis. In recent years, plasma exchange has been highly recognized for the treatment of IAE.^26^, ^27^ There were only 4 patients treated with plasma exchange according to severity of illness and requested by the patient's guardian. As mentioned above, the treatment value of antivirals, corticosteroids, IVIG, and plasma exchange for IAE patients needs to be further studied.

Influenza vaccination is an important tool to reduce and prevent influenza-related complications and mortality. A study by Ferdinands et al. found full influenza vaccinations could reduce 74 % of PICU admissions.^29^ Another study from the United States also reported an influenza vaccine effectiveness of 65 % in preventing laboratory-confirmed influenza-associated death among children.^30^ In China, influenza vaccination is recommended for children ≥ 6 months and < 5 years of age but not funded.^31^ In this study, only one patient received seasonal influenza vaccination. Effective measures should be taken to increase vaccination coverage for children in China.

There were several limitations to the present study. First, as a retrospective study, information bias was unavoidable. Second, this was a single-center study, which may limit the generalizability of these results. Third, due to the relatively small sample size, multivariable analysis was not used to identify risk factors of poor outcome.

In conclusion, we have found that a significant proportion of influenza-related hospitalized patients can suffer from IAE, and IAE can lead to a high mortality rate in children. We also found that lower Glasgow coma score, longer duration of fever, with underlying medical conditions and complications pose the greatest risk to poor prognosis. The present findings emphasize the importance of vaccination to reduce the risks of influenza infection in children. Further larger multicenter studies are required to evaluate the potential risk factors for poor outcomes of IAE.

## CRediT authorship contribution statement

**Min Yang:** Visualization, Writing – original draft. **Ling Yi:** Formal analysis, Data curation, Writing – original draft. **Fenglin Jia:** Investigation. **Xiaobin Zeng:** Investigation. **Zhongqiang Liu:** Visualization, Investigation, Writing – review & editing.

## Declaration of competing interest

The authors declare no conflicts of interest.
